# Machine learning-enabled detection of attention-deficit/hyperactivity disorder with multimodal physiological data: a case-control study

**DOI:** 10.1186/s12888-024-05987-7

**Published:** 2024-08-05

**Authors:** Dimitrios Andrikopoulos, Georgia Vassiliou, Panagiotis Fatouros, Charalampos Tsirmpas, Artemios Pehlivanidis, Charalabos Papageorgiou

**Affiliations:** 1 Feel Therapeutics Inc., 479 Jessie St., San Francisco, CA94103 CA USA; 2 First Department of Psychiatry, Eginition Hospital, Medical School National and Kapodistrian University of Athens, Athens, Greece; 3 Neurosciences and Precision Medicine Research Institute “Costas Stefanis”, University Mental Health, Athens, Greece

**Keywords:** Precision medicine, Data-driven diagnostics, Attention-deficit/hyperactivity disorder, Psychophysiological data, Electrodermal activity, Heart-rate variability, Skin temperature

## Abstract

**Background:**

Attention-Deficit/Hyperactivity Disorder (ADHD) is a multifaceted neurodevelopmental psychiatric condition that typically emerges during childhood but often persists into adulthood, significantly impacting individuals’ functioning, relationships, productivity, and overall quality of life. However, the current diagnostic process exhibits limitations that can significantly affect its overall effectiveness. Notably, its face-to-face and time-consuming nature, coupled with the reliance on subjective recall of historical information and clinician subjectivity, stand out as key challenges. To address these limitations, objective measures such as neuropsychological evaluations, imaging techniques and physiological monitoring of the Autonomic Nervous System functioning, have been explored.

**Methods:**

The main aim of this study was to investigate whether physiological data (i.e., Electrodermal Activity, Heart Rate Variability, and Skin Temperature) can serve as meaningful indicators of ADHD, evaluating its utility in distinguishing adult ADHD patients. This observational, case-control study included a total of 76 adult participants (32 ADHD patients and 44 healthy controls) who underwent a series of Stroop tests, while their physiological data was passively collected using a multi-sensor wearable device. Univariate feature analysis was employed to identify the tests that triggered significant signal responses, while the Informative k-Nearest Neighbors (KNN) algorithm was used to filter out less informative data points. Finally, a machine-learning decision pipeline incorporating various classification algorithms, including Logistic Regression, KNN, Random Forests, and Support Vector Machines (SVM), was utilized for ADHD patient detection.

**Results:**

Results indicate that the SVM-based model yielded the optimal performance, achieving 81.6% accuracy, maintaining a balance between the experimental and control groups, with sensitivity and specificity of 81.4% and 81.9%, respectively. Additionally, integration of data from all physiological signals yielded the best results, suggesting that each modality captures unique aspects of ADHD.

**Conclusions:**

This study underscores the potential of physiological signals as valuable diagnostic indicators of adult ADHD. For the first time, to the best of our knowledge, our findings demonstrate that multimodal physiological data collected via wearable devices can complement traditional diagnostic approaches. Further research is warranted to explore the clinical applications and long-term implications of utilizing physiological markers in ADHD diagnosis and management.

## Introduction

Attention Deficit Hyperactivity Disorder (ADHD) is a multifaceted neurodevelopmental psychiatric disorder typically emerging during childhood. Individuals with ADHD exhibit distinct personality traits and cognitive characteristics that impede their ability to regulate attention, behavior, and emotional responses [1]. The symptoms often manifest themselves as a persistent challenge to maintain focus, accompanied by hyperactivity or impulsivity, resulting in impairments in cognition and various life domains, including social, educational, and vocational among others [2, 3]. Although ADHD primarily arises early in childhood, in most cases, it often persists into adulthood, exerting long-term effects on individuals’ functioning, productivity, and overall quality of life [4, 5]. Although extensive research efforts have focused on childhood ADHD, where approximately 8% of children and 6% of adolescents worldwide are affected [6], researchers have only recently started investigating the disorder in adults [7–11], with an estimated global prevalence of 6.76% as reported by a 2022 study [12].

Although increased self-control in adulthood can help mitigate some of the symptoms associated with hyperactivity, difficulties in regulating emotional responses, maintaining focus, inferiority and feelings of impulsivity often persist beyond childhood [5, 13]. Inattention in adults with ADHD may lead to a slower pace of thinking and decision-making, as they often become entangled in irrelevant details [14]. Meanwhile, hyperactivity is linked to an inner sense of restlessness, with symptoms such as talking too much or too loudly, pacing up and down, or even experiencing muscle strain when seated [14]. Overall, adults with ADHD encounter several challenges in their personal, social, academic and vocational lives [2, 14, 15]. Their struggle to maintain focus and complete tasks renders them less productive at work [16], leading to work loss and substantial economic repercussions [17]. Furthermore, adults with ADHD are more prone to addictive behaviors (e.g. substance abuse disorders) [18, 19], while they also have a higher risk of injury and serious accidents, particularly stemming from risky driving behavior [20, 21].

In light of the profound impact ADHD has on individuals throughout their lives, diagnosing ADHD in adults is a crucial step towards managing and treating the disorder, ultimately enhancing their overall well-being and quality of life. The current standard for diagnosing ADHD in adults relies on a combination of psychometric questionnaires, in-person interviews with the individual and/or their parents to gather a comprehensive clinical history, and clinical assessments to identify the presence of specific symptoms [22]. Although widely used, this process has several limitations that can significantly affect its overall effectiveness [1]. Firstly, the current diagnostic process primarily relies on face-to-face assessments, which presents a significant limitation in terms of accessibility. Additionally, it can be time-consuming and economically burdensome, as multiple sessions may be required [22]. Furthermore, obtaining ancillary information to establish ADHD onset in childhood, which in most cases extends to adulthood, relies on subjective recall and may not always be available [23]. Moreover, recognizing the occurrence of symptoms is subject to the clinician’s interpretation, introducing potential bias into the diagnosis [24, 25]. Finally, ADHD symptomatology does not uniquely correlate with an ADHD condition and can overlap with symptoms of other psychiatric conditions [26]. It is estimated that approximately three out of four adults with ADHD suffer from at least one additional mental disorder such as depression, anxiety, personality disorders, or substance abuse [14, 24, 25]. The non-distinct nature of ADHD symptoms and the presence of psychiatric comorbidities introduce an additional level of uncertainty and complexity into the current diagnostic process [24, 26, 27].

In an effort to address some of the limitations associated with the standard of care interview-based diagnostic approaches, clinical experts working with ADHD patients have sought additional tools to provide objective data and facilitate more informed decisions [28, 29]. In this context, evaluations of the neuropsychological and neurophysiological aspects of the disorder have emerged as promising tools, though they have attracted varying levels of scientific interest. On the one hand, neuropsychological evaluations, which are more popular, have been employed to uncover impairments in various cognitive functions [29], serving as objective indicators of ADHD [28]. One such evaluation method is the Continuous Performance Tests (CPTs), which measure sustained attention and vigilance in a task-oriented computerized setting [1, 28, 30, 31]. Additionally, Stroop tests are commonly utilized to assess selective attention, interference and inhibitory control [31–34]. While the value of CPTs has been demonstrated in child populations [35, 36] and several studies [37–39] have highlighted the potential of Stroop tests for adults with ADHD, the utility of neuropsychological evaluations for diagnosing ADHD in adults is limited, as conclusive results cannot always be obtained [28, 29, 40–43]. The primary drawback of this approach is that it targets specific cognitive deficits, which may fail to capture impairments when multiple cognitive domains are affected, as is often the case for ADHD [28, 44].

On the other hand, clinicians have recently focused on the neurophysiological impact of the disorder, including brain and Autonomic Nervous System (ANS) functioning [5, 45–51]. Research has shown that ADHD is associated with alterations in brain function that may lead to cognitive impairment [49]. These alterations can be captured by imaging techniques capable of measuring functional brain activity (e.g. Electroencephalography (EEG), Magnetoencephalography, and Magnetic Resonance Imaging (MRI)) [52–57]. Recent studies [52–57] have underscored the correlation between functional brain activity, primarily captured by Functional Magnetic Resonance Imaging and EEG methods, and an underlying ADHD condition. However, such methods can not be used for large-scale testing and deployment, as MRI and EEG setups tend to be expensive, time-consuming, and obtrusive data collection approaches. Consequently, integrating these methods into current clinical practice may prove inefficient and impractical [58].

Besides its impact on brain function, studies have demonstrated that ADHD also affects the functioning of the Autonomic Nervous System (ANS) [50], which controls involuntary physiological processes [59, 60]. Within the context of ADHD, several studies [5, 46–48, 50] have suggested dysregulation in the part of ANS responsible for controlling arousal. This dysregulation may be linked to the behavioral challenges experienced by individuals with ADHD [47, 50]. Arousal of the ANS can be gauged in real-time by measuring physiological data [61], such as electrodermal activity (EDA), heart rate (HR), heart rate variability (HRV) and skin temperature (ST) [13, 62–66]. Several studies have proposed that these data modalities [45, 67–75] may carry useful information and offer valuable insights into an underlying ADHD condition. However, there has been limited focus on adult ADHD [45, 67–70, 72], with most studies involving relatively complicated setups [45, 68, 71–75] that hinder scalability and fail to leverage wearable sensors that enable multimodal data capturing in an unobtrusive and continuous manner [58].

In summary, neuropsychological and neurophysiological evaluations provide complementary and valuable information, offering different insights into ADHD conditions. Both approaches appear beneficial for clinicians and have the potential to address the limitations and complement the current symptom-based diagnostic process for adults with ADHD. Given that that research has demonstrated that ADHD is characterized by dysregulation in controlling physiological arousal, exploring the distinct physiological expressions of adult ADHD patients during potentially arousing conditions, such as neuropsychological tests, is of particular interest. Neuropsychological tests can reliably elicit ANS responses related to attention, cognitive control, and emotional regulation [76–80]. Therefore, the main focus of this study is to assess the potential capability of physiological data (i.e. EDA, HRV, ST) to distinguish ADHD adults, when collected during a series of neuropsychological evaluations (i.e. Stroop tests).

The selection of these specific data modalities serves multiple purposes. Firstly, we aim to explore the potential of physiological markers as standalone indicators for ADHD. To the best of our knowledge, there is limited research exploring the connection between physiological data and adult ADHD compared to neuropsychological assessments. Secondly, we seek to assess the utility of unobtrusively and continuously collected data, using scalable wearable technology. Such approaches would be more feasible for real-world applications and more likely to be integrated into current clinical practice compared to brain activity evaluations. Lastly, even though including additional data modalities (e.g. metrics from neuropsychological evaluations) in our analysis would be interesting, at the same time, it would significantly increase the complexity and dimensionality of our models, potentially compromising the robustness of the results and necessitating a more extensive dataset.

Therefore, this study employs EDA, HRV and ST data collected from a wrist-worn sensor during neuropsychological evaluations to investigate their potential utility in the diagnostic process for adult ADHD. For this purpose, we leverage the Feel Digital Precision Medicine Platform (DPMP), which enables continuous, real-time, and unobtrusive data capturing through a single wearable device. Furthermore, the platform offers signal processing and feature extraction capabilities, which are also utilized in this work. The main aims of this work are to: (i) evaluate the feasibility and utility of integrating physiological data, captured through a cost-effective and unobtrusive wearable sensor, into the current diagnostic framework for adult ADHD, (ii) develop and validate the performance and diagnostic capability of a machine learning (ML)-based pipeline in distinguishing ADHD adults, leveraging our DPMP and (iii) assess the complementary value of physiological data in providing a comprehensive understanding of ADHD symptoms.

## Methods

### Study design and participant recruitment

This was an observational, case-control study that included two groups of participants, an experimental and a control one. Participants for this study were recruited in collaboration with the First Department of Psychiatry of the Athens School of Medicine. The experimental group (EG) consisted of adult patients diagnosed with ADHD who were evaluated by healthcare professionals of the clinic. The assessment process for potential candidates in the EG included completing a questionnaire that consisted of questions collecting demographic, educational, occupational, and clinical data. This was followed by a battery of screening instruments, including a modified version of the Barkley Adult ADHD Rating Scale (BAARS) [81], the Autism-Spectrum Quotient (AQ) [82] and the Empathy Quotient (EQ) [83, 84]. Additionally, the semi-structured Diagnostic Interview for ADHD in Adults (DIVA) was administered to all patients [85, 86]. Complementary information was also collected from relatives. The second step of the assessment process entailed a comprehensive two-hour psychiatric examination by an experienced psychiatrist in the Psychiatry Department. This examination aimed to explore the presence of lifetime psychopathology using the Mini-International Neuropsychiatric Interview (M.I.N.I.) Greek version [87], which consists of a short structured interview assessing patient symptoms and signs against diagnostic criteria outlined in the Diagnostic and Statistical Manual of Mental Disorders (DSM-5) [3]. Overall, the main inclusion criteria for the EG candidates were: (i) ADHD diagnosis; (ii) age$\ge$18 years; (iii) IQ>70; (iv) proficient in Greek; and (v) willing and able to give informed consent for participation. On the other hand, the main exclusion criteria were: (i) major psychiatric disorder (e.g., schizophrenia, bipolar disorder); (ii) significant neurological conditions (e.g., epilepsy, traumatic brain injury); and (iii) severe learning disabilities. The final diagnosis regarding the presence of ADHD was based on DSM-5 criteria. Furthermore, candidates for the control group (CG) included neurotypical adults who had not undergone any screening for the presence of ADHD. Control participants were required to meet the same inclusion and exclusion criteria with EG participants, apart from the ADHD diagnosis.

**Fig. 1:**
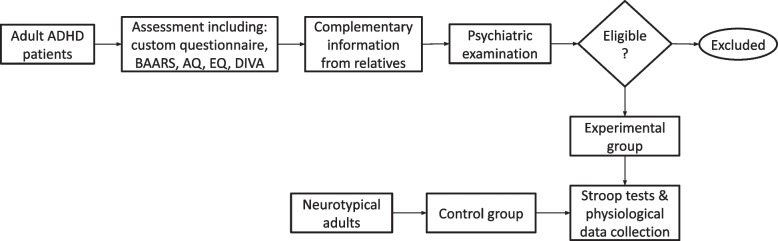
Flowchart for participant screening and recruitment

Eligible candidates for both groups were then informed about the study scope, aims, and experimental process and were invited to participate. Those who expressed interest in joining had to select their preferred time slot and schedule an in-person experimental session. During this session, held at the First Department of Psychiatry of the Athens School of Medicine, participants were provided the Feel physiological data monitoring device (see Data acquisition, preprocessing and feature extraction infrastructure section). This device unobtrusively and continuously collected participants’ data, as they progressed through the various steps of experimental protocol. The flowchart of the participant screening and recruitment process is illustrated in Fig. 1.

### Experimental setup

The experimental protocol for both the EG and CG, consisted of a series of six computerized Stroop tests during which participants were required to answer as quickly and accurately as possible: three color-word Stroop tests, two number Stroop tests, and one emotion Stroop test. At the same time, their physiological data was collected via the Feel Monitoring Device. The Stroop tasks were developed in Python programming language [88] and were presented using a Windows personal computer and a 17-inch flat screen. Participants provided their responses for the number and emotion Stroop tests using the computer keyboard, while the number of correct and wrong responses for the color-word Stroop tests were manually recorded by the researcher executing the experiment. The start and end times for each test were manually input by the researcher through the data collection mobile application (see Data acquisition, preprocessing and feature extraction infrastructure section). This ensured that the physiological data recorded by the wearable device was synchronized with the task events. In more detail, each test was designed as follows:

- *Color-Word Stroop*: This step consisted of three different tests labeled as C1, C2 and C3. In C1, participants were instructed to name a color word depicted in a neutral color (e.g. the word “red” presented in a black-colored font). In C2, participants were presented with differently colored symbols and asked to identify the color of the font (e.g. symbol XXX presented in a blue-colored font). In C3, color words were depicted in a different color, which the subjects were asked to identify, ignoring the word itself (e.g. the word “red” presented in a blue-colored font). Each of the color-word Stroop tests lasted approximately one minute, during which participants were prompted to give as many answers as possible from a list of 200 words. The total duration of the session was 3 minutes.
- *Number Stroop*: This part consisted of two tests labeled as N-S (Number-Size) and N-V (Number-Value). In both tests, participants were presented with pairs of numbers, each one displayed in a different font size. In the congruent condition, the number with the higher value was displayed in the larger font size, while in the incongruent condition, the number with the higher value was displayed in the smaller font size. During the N-S test, participants were asked to identify the number with the larger font size, while in N-V, they were asked to identify the number with the larger value. Each test included 100 pairs of numbers. This series of tests did not have a fixed duration and test duration depended on the time required by each participant to respond to all pairs of numbers. The average duration for each test was 3.9 minutes, with the total duration of this session being on average 7.8 minutes.
- *Emotion Stroop*: This part consisted of a single test labeled as E. In the Emotion Stroop task, participants were instructed to name as quickly and accurately as possible the color of the presented words, while ignoring their meaning, which could be either neutral (e.g. tower, fork, etc.) or negative (e.g. crime, traitor, annoying, etc.). This session included 30 neutral and 30 negative words, presented in 4 different colors. The total number of trials was 240. The number of words displayed was fixed, and like the Number Stroop step, the total test time varied among participants. The duration of this session was on average 4.3 minutes.

### Data acquisition, preprocessing and feature extraction infrastructure

**Fig. 2:**
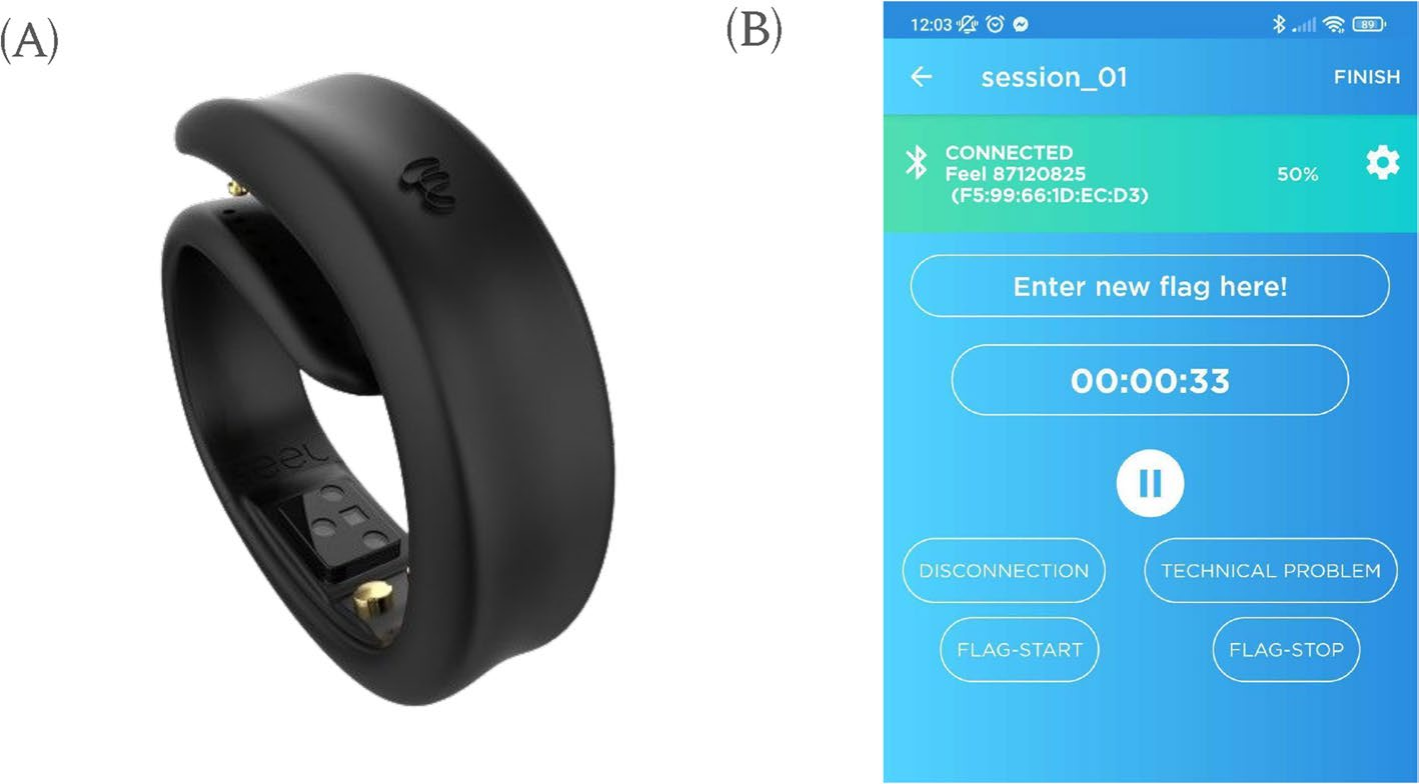
The feel monitoring device (**A**) and the feel mobile app (**B**)

For the purposes of this study, we have leveraged the capabilities and functionalities of our proprietary DPMP. The DPMP is a remote patient monitoring platform designed for unobtrusive, passive, and continuous monitoring and analysis of neurological and psychiatric patient data. The platform facilitates the collection, curation, and processing of multimodal data, with a focus on the discovery, extraction, and validation of metrics and biomarkers for various use cases in the fields of neurology and psychiatry. In this study, the following components of the platform were employed:

- *Feel Monitoring Device*: The wrist-worn device features five embedded sensors and connects to the user’s smartphone via Bluetooth to accommodate continuous and passive data collection and transmission (Fig. 2A). The device collects psychophysiological, activity, and ambient conditions data, which have proven to be relevant and highly valuable for many neurological and psychiatric applications [89–91]. More specifically, the following data modalities are captured by the wearable device: EDA, HR, HRV, ST, 9-axis Inertial Measurement Unit, ambient temperature, and humidity. The focus of this work is on the first three data modalities.
- *Feel Mobile app*: The Mobile app (Fig. 2B) connects to the Feel Monitoring Device, collects data, and transfers it from the device to the secure cloud-based processing infrastructure. The app allows users to control the start/stop of the data acquisition session and annotate specific timestamps during the acquisition with custom labels, indicating the start and end of each session step. These labels are used to identify data segments associated with each Stroop test. The Feel Mobile App is available for Android and iOS platforms.
- *Digital Endpoints Development & Biomarker Discovery Infrastructure*: This sophisticated infrastructure integrates data curation, signal processing, time series analysis, and pattern recognition tools and frameworks for noisy artifact detection and denoising, signal annotation and segmentation, and feature extraction purposes. The processing pipeline for collected time series commences with the denoising step, where noisy signal parts are identified and the impact of noise artifacts is appropriately mitigated. Subsequently, time segments of the collected signals corresponding to each of the six Stroop tests are identified, using the timestamp annotations that have been inserted from the Feel mobile app. Finally, a wide variety of proprietary features are calculated for each segment of the acquired time series. These features range from simple statistical metrics to more complex and highly nonlinear ones reflecting the morphological, frequency, repeatability, and predictability characteristics of the signals. Specifically, 103 features have been extracted from the EDA signals, 77 from the HRV signals, and 11 from the ST signals, resulting in a feature set of 191 features available for our analysis.

### Statistical analysis and decision pipeline

**Fig. 3:**
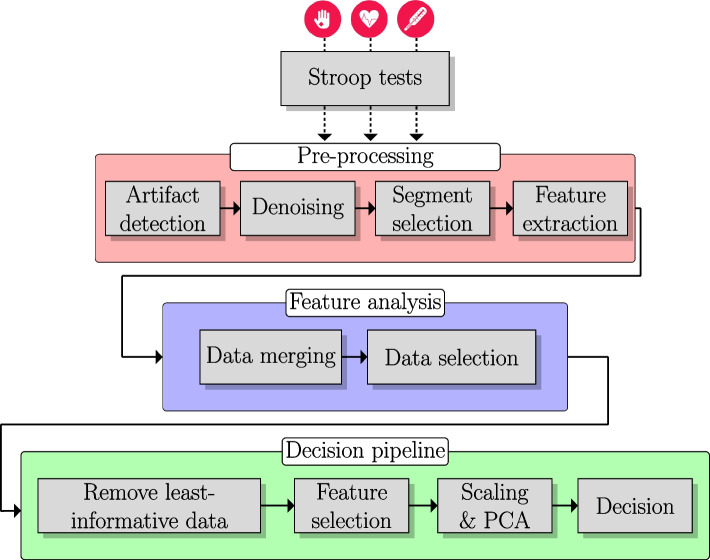
Overview of the data processing and decision pipeline

We employed descriptive statistics to analyze the demographic characteristics of the two groups. Additionally, we compared the age distribution of the two groups using an independent t-test [92] and assessed the gender ratio using the $\chi ^2$ test [93]. Leveraging the features extracted by the Biomarker Discovery infrastructure, we conducted a two-step univariate feature analysis to identify the best subset of the collected data that, when fed into an ML pipeline, would be able to distinguish between the experimental and control groups. An overview of the data processing and decision pipeline is shown in Fig. 3.

Firstly, within each group (i.e. EG and CG), we investigated the variance of each feature at the individual level. To accomplish this, we performed a series of nonparametric pairwise statistical tests for every pair of Stroop tests using the Wilcoxon signed-rank test [94]. Utilizing this method, a univariate analysis was performed in order to determine the proportion of total features showing a statistically significant difference between pairs of Stroop tests. A low ratio suggests minimal impact at the individual level for the specific combination of tests, allowing paired data to be treated as uncorrelated. Consequently, the feature data from two Stroop tests could be combined and analyzed together under a common test label. The second step of our statistical analysis involved identifying the data subsets that yielded more profound differences across features between the two groups (CG and EG). To achieve this, for each type of test (either the original tests or the bundled ones determined by the first level of our analysis), we conducted a univariate feature analysis using Kolmogorov-Smirnov statistical tests [95]. Subsequently, the subset of our dataset corresponding to the Stroop tests that yielded a higher percentage of features with statistically significant differences between the two groups were selected as inputs to the learning algorithms. For both levels of the statistical analysis, the cutoff *P*-value used for statistical significance was set at 0.1. For both statistical tests, we utilized the corresponding implementations in the SciPy Python package [96].

Having built a more informative data subset, the decision pipeline consists of three main parts: i) informative point selection, ii) feature selection, and iii) classification. The input of this pipeline is a dataset that includes the features identified as the most informative from the previously discussed statistical analysis. Separate datasets were constructed both for each of the three signals (i.e. EDA, HRV, and ST) and for their fusion. In the latter case, the feature space is constructed by concatenating the features from all signals.

#### *Informative point selection*

We enhance the separability of the two groups by identifying and discarding the least informative points [97]. Towards this, we utilize a modified version of the k-Nearest Neighbors algorithm (KNN), namely the Informative KNN (i-KNN) [98]. The least informative points are defined as data points that are (isolated) instances of one class in a *n*-dimensional feature space that reside in a neighborhood with a high density of points from the opposite class. For each data point $\textbf{x}_i$ with class $y_i$ we select *k* nearest neighbors and calculate for each neighbor $\textbf{x}_j$, the associated informativeness given by Eq. (1) [98]:

1 $$\begin{aligned} P\left( \textbf{x}_j|\textbf{x}_i\right) =\frac{1}{C_i}d\left( \textbf{x}_j,\textbf{x}_i\right) ^{\eta \left( \textbf{x}_j,\textbf{x}_i\right) }\Lambda \left( \textbf{x}_j\right) ^{1-\eta \left( \textbf{x}_j,\textbf{x}_i\right) } \end{aligned}$$

where *d* is a distance function defined as $d(\textbf{x}_j,\textbf{x}_i)=e^{-|\textbf{x}_j-\textbf{x}_i|^2}$, $\eta$ represents the ratio of neighbors that have the same class $y_j$ as $\textbf{x}_j$ and $C_i$ is a normalization factor such that $\sum \nolimits _j P(\textbf{x}_j|\textbf{x}_i)=1$. The function $\Lambda (\textbf{x}_j)$ can be interpreted as a weighting parameter, which quantifies how far apart the point $\textbf{x}_j$ lies from the rest of the $k-1$ neighbors of $\textbf{x}_i$ which have a different label than $y_j$. This weighting parameter is defined in Eq. (2).

2 $$\begin{aligned} \Lambda \left( \textbf{x}_j,\textbf{x}_i\right) \prod \limits _{n=1}^{k}\left( 1-d\left( \textbf{x}_j,\textbf{x}_n\right) \left( 1-\delta _{y_j,y_n}\right) \right) \end{aligned}$$

with $\delta _{y_j,y_n}$ being the Kronecker delta which equals 1 only if $y_j=y_n$ and 0 otherwise. We rank the *k* neighbors according to their informativeness (Eq. (1)) and classify $\textbf{x}_i$ using the majority vote of the labels from the *M* neighbors with the highest informativeness. This classification decision is denoted as $\tilde{y}_i$. We perform this process for $k\in \{3,5,7,10\}$ and for each *k*, we use $M\in [1,k]$ (25 possible combinations in total). For each value *l* for *k* and *m* for *M*, we compare the real label $y_i$ with the decision $\tilde{y}_i$ from the most informative neighbors and assign a binary score $s(\textbf{x}_i,l,m) = 1-\delta _{y_i,\tilde{y}_i}$. We rank every vector $\textbf{x}_i$ using the average $\bar{s}(\textbf{x}_i)$ of the scores $s(\textbf{x}_i,l,m)$, which is defined as shown in Eq. (3). A higher value of $\bar{s}(\textbf{x}_i)$ implies that $\textbf{x}_i$ is less informative for its corresponding class $y_i$ and could thus be discarded. In this study, we examined two input datasets to the decision pipeline: i) the full dataset and ii) the reduced dataset after discarding the worst 5% of the data points, as described above. The implementation of this algorithm was performed in Python utilizing the respective packages [99].

3 $$\begin{aligned} \bar{s}(\textbf{x}_i)=\frac{1}{25}\sum \limits _{l=1}^{l=k}\sum \limits _{m=1}^{m=k}s(\textbf{x}_i,l,m) \end{aligned}$$

#### Feature selection

Regarding feature selection, we utilize the relative Median Absolute Deviation (rMAD, Eq. (4)) and the relative Interquartile Range (rIQR, Eq. (5)) as lower bounds to discard low-variance features that have minimal impact on the separability of the two groups. We have selected these two quantities as thresholds since they are robust to outlier values.

In Eqs. (4) and (5), $\textbf{f}_i$ refers to a feature vector, i.e. a list of the values of the feature *i*. Furthermore, $\text {med}(\textbf{f}_i)$ in Eq. (4) is the median and $p(\textbf{f}_i,q)$ in Eq. (5) represents the *q*-th percentile of $\textbf{f}_i$.

4 $$\begin{aligned} \text {rMAD}\left( \textbf{f}_i\right) =\frac{\text {med}\left( |\textbf{f}_i-\text {med}\left( \textbf{f}_i\right) |\right) }{\text {med}\left( \textbf{f}_i\right) } \end{aligned}$$

5 $$\begin{aligned} \text {rIQR}\left( \textbf{f}_i\right) =\frac{\left| p\left( \textbf{f}_i,0.75\right) -p\left( \textbf{f}_i,0.25\right) \right| }{p\left( \textbf{f}_i,0.25\right) } \end{aligned}$$

Both rMAD and rIQR have been normalized respectively. For rIQR if $p(\textbf{f}_i,0.25)=0$, then $p(\textbf{f}_i,0.75)$ is used in the denominator.

#### Classification

For the classification step, we first utilize Principal Component Analysis (PCA) to construct linear combinations of the features that best capture the variability, and thus the useful information, of the dataset. Then, we employ four widely used classification algorithms: Logistic Regression (LR), KNN, Random Forests (RF) and Support Vector Machines (SVM) [99]. For each algorithm, a set of classifier-specific hyperparameters along with the rMAD and rIQR thresholds are tuned to achieve the best accuracy. This tuning process is conducted using a 100-fold cross-validation scheme, where we split the data into a 70%-30% train-test stratified split. It is noteworthy that whether the full or reduced dataset was used was not treated as a hyperparameter, ensuring that each algorithm utilizes the same data points across all validation folds. For each input dataset, we have the option to keep all input data, or discard the least informative points and evaluate the output of the decision pipeline on the obtained accuracy, sensitivity and specificity of the test sets.

### Ethical considerations

This observational study was conducted in accordance with the Declaration of Helsinki and was approved by the Ethics Committee of the National and Kapodistrian University of Athens, 1st Department of Psychiatry, Eginition Hospital. Prior to their participation in the study, all individuals were fully informed about the study scope, objectives, methodology, and components, and they provided written informed consent. They were also informed that their participation was voluntary and they could withdraw from the study at any time. Each participant was assigned a unique identifier upon providing informed consent. The collected data was pseudonymized, and no personal identifiers were used during data processing and reporting of results. Furthermore, participants did not receive any monetary compensation for their involvement in the study.

## Results

### Sample characteristics

A total of 95 individuals participated in this study, with 58 in the control group and 37 in the ADHD group. There were no difference in the age ($35.18\pm 11.14$ vs $32.58\pm 11.39$, $t(93)=1.06$, $P=.29$) and gender ratio (Male/Female, 32/26 vs. 24/13, $\chi ^2(1, 95)=0.5221$, $P=.46$) between the two groups. Out of the 95 participants who joined the study, 4 did not complete the experimental process due to time limitations or personal discomfort issues. Additionally, physiological data was not retrievable for 15 participants, mainly due to protocol execution errors, mobile app malfunctions, and internet connectivity issues. The remaining participants (i.e. 44 CG and 32 EG participants) successfully completed the experimental process. For this group of participants, there were no significant differences in the age distributions (36.23 ± 9.36 vs. 33.26 ± 12.18, $t(74)=1.18$, $P=.24$), as well as for the gender ratio (Male/Female 29/15 vs. 21/11, $\chi ^{2}(1,76)<10^{-4}$, $P=.97$). Finally, 23 out of 32 EG participants were receiving ADHD medication.

### Feature analysis

**Fig. 4:**
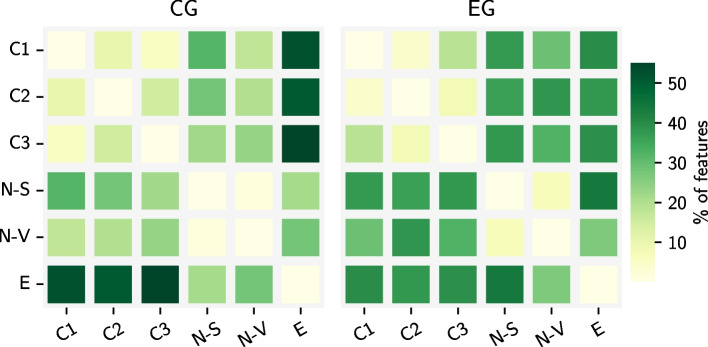
Percentage of features that differ significantly ($P\le .1$) within the control (CG; left) and experimental (EG; right) groups, per every couple of Stroop Tests

Firstly, we explore the variability of the extracted features within the CG and EG for all combinations of Stroop tests in the experimental process, employing the Wilcoxon signed-rank test. In Fig. 4, we showcase the ratio of the total features that show a statistically significant change between every pair of tests for the CG and EG in the left and right plots, respectively. The *x* and *y*-axis in each plot indicate the two tests involved in the comparison. For example, the element in the first row and second column corresponds to the output of the comparison between the C1 and C2 Stroop tests. Accordingly, the elements on the diagonal are all equal to 0, since these correspond to the output of the comparison of a Stroop test with itself. It becomes evident that a clear pattern exists involving Stroop tests of the same type (i.e. Color-Word, Number, or Emotion). For such cases, the percentage of features showing a statistically significant change between the corresponding Stroop tests within both CG and EG is much lower (light-colored regions) than for cases with Stroop tests of different types being compared (dark-colored regions). Therefore, data from tests C1, C2, and C3, as well as N-Size and N-Value, can be bundled together under the new label “C” and “N”, respectively.

**Fig. 5:**
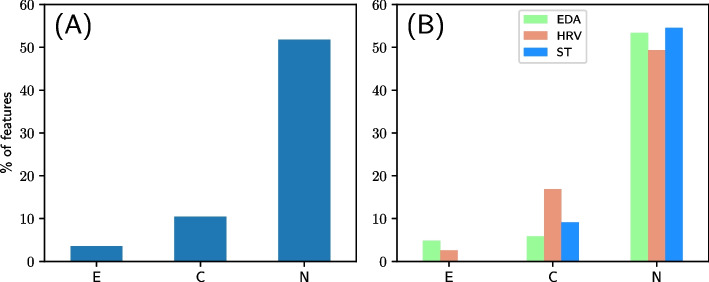
Percentage of features showing statistically significant differences ($P\le .1$) between the control (CG) and experimental (EG) groups for the Emotion (E), Color-Word (C), and Number (N) Stroop tests in total (**A**) and per data modality (**B**)

After bundling together the Color-Word and Number Stroop tests, we now have three types of tests: Color (C), Number (N), and Emotion (E). As the next step of the analysis, we explore how much the extracted features differ between the CG and EG for the three types of tests. For each case, the percentage of total features that showed a statistically significant difference between the CG and EG is illustrated in Fig. 5A. Moreover, the respective percentages per data modality (i.e. EDA, HRV and ST) are shown in Fig. 5B. As can be seen, the dataset consisting of N tests shows a much larger percentage of features differing between the two groups compared to the C and E tests, with more than half of the features being significantly different. Therefore, in the rest of this work, we focus solely on the subset of our dataset that includes features only from the N test.

### Decision pipeline

For the evaluation of the performance of the decision pipeline, three metrics have been utilized: accuracy, sensitivity, and specificity. The former corresponds to the ratio of the correctly identified instances (true CG + true EG) to the total number of instances and reflects how many instances were correctly classified in total. Sensitivity assesses the capability of the model to correctly identify positive (true EG) instances and is calculated as the ratio of correctly identified EG instances to their total number. Similarly, specificity evaluates the same aspects for the CG instances and is extracted as the ratio of correctly identified CG instances to their total number. Tables 1, 2, 3 and 4 present these metrics for the different classification models employed for each physiological signal (i.e. EDA, HRV, ST) and their fusion, utilizing data captured during the Number Stroop tests. In all cases, the results for both the full and the reduced datasets are provided.

#### Electrodermal activity (EDA)

Table 1 displays the accuracy, sensitivity, and specificity of different classification models using both the original and reduced feature sets from EDA data. It should be noted that in most cases, all metrics improve when the least informative points are discarded, and the reduced dataset is utilized. More specifically, all performance metrics improve for the LR and RF algorithms, while only accuracy and sensitivity are enhanced for KNN and SVM. Notably, for SVM, the capability to identify CG instances is significantly reduced (specificity=40%). Interestingly, noisy data points appear to play a crucial role in constructing the support vectors, as the performance of the SVM model in terms of sensitivity and specificity is reversed when these points are discarded. The LR model achieves the highest accuracy and sensitivity but performs relatively poorly in identifying CG data points, with specificity below 60%. On the other hand, the RF classifier achieves a more balanced performance, with all performance metrics ranging from 70% to 74%.

**Table 1:** Accuracy, sensitivity and specificity for the LR, RF, KNN and SVM classification models using the original and reduced feature set from EDA data

	Method							
	LR		RF		KNN		SVM	
Metric	*Original*	*Reduced*	*Original*	*Reduced*	*Original*	*Reduced*	*Original*	*Reduced*
*Accuracy*	65.8%	73.5%	62.3%	72.6%	58.3%	68.5%	56.5%	60%
*Sensitivity*	82.5%	86.4%	66%	74.6%	54.8%	76.1%	16.6%	77.7%
*Specificity*	51.6%	58.8%	59.2%	70.5%	61.2%	59.8%	90.4%	40%

#### Heart rate variability (HRV)

Similar to the previous case, the performance of all models is generally enhanced when the most informative data points are retained (Table 2). This becomes also evident for the SVM case, at least with regard to the accuracy and sensitivity metrics, which are significantly improved. This time, the KNN algorithm achieves the most balanced performance, with all metrics hovering around 78%. The RF algorithm also demonstrates a balanced performance, albeit with slightly lower metrics ranging from 73% to 77%. On the other hand, the capability of the LR model to identify the CG instances is the best among all models with a specificity of 82.2%, while also showing similar performance to the RF and KNN models with respect to the other metrics. Finally, the SVM achieves the best sensitivity, which is close to that of the KNN, but shows poorer performance in identifying CG data points. Similar to the EDA case, the SVM is notably influenced by the presence of the least informative data points.

**Table 2:** Accuracy, sensitivity and specificity for the LR, RF, KNN and SVM classification models using the original and reduced feature set from HRV data

	Method							
	LR		RF		KNN		SVM	
Metric	*Original*	*Reduced*	*Original*	*Reduced*	*Original*	*Reduced*	*Original*	*Reduced*
*Accuracy*	67.4%	77.6%	62.7%	75.6%	66.1%	78%	56.8%	71.6%
*Sensitivity*	67.2%	73.8%	61.5%	77.3%	64.3%	77.5%	36.1%	78.1%
*Specificity*	67.5%	82.2%	63.7%	73.5%	67.7%	78.7%	75.3%	63.6%

#### Skin temperature (ST)

In the case of the ST signal (Table 3), the LR algorithm achieves an accuracy of 72% with a sensitivity of 89%. However, there is a notable increase in misclassification of CG participants, with a specificity close to 55%. The KNN and RF models exhibit similar performance, with more balanced metrics compared to the other models. On the other hand, the SVM algorithm demonstrates the least balanced performance, successfully identifying most of the EG data (sensitivity: 85%) but misclassifying the majority of CG data (specificity: 40%). Retaining the most informative data points affects the performance of all algorithms, with the SVM being particularly impacted.

**Table 3:** Accuracy, sensitivity and specificity for the LR, RF, KNN and SVM classification models using the original and reduced feature set from ST data

	Method							
	LR		RF		KNN		SVM	
Metric	*Original*	*Reduced*	*Original*	*Reduced*	*Original*	*Reduced*	*Original*	*Reduced*
*Accuracy*	67.3%	72%	64.5%	71.6%	65.6%	71.7%	57.9%	63.9%
*Sensitivity*	81.9%	89.4%	68%	79.9%	63.4%	79.9%	27.9%	85.8%
*Specificity*	54.8%	53.6%	61.6%	62.7%	67.4%	62.9%	83.5%	40.7%

#### Data modalities fusion

Finally, when fusing data from all physiological signals together, we effectively expand the feature space and utilize the information from each signal simultaneously. As shown in Table 4, this leads to improved performance for all classifiers. Interestingly, the SVM classifier, which exhibited the least balanced performance when each signal was utilized separately, now shows almost identical capability to identify both the CG and EG data well, with sensitivity and specificity close to 82%. The rest of the classifiers demonstrate similarly balanced performance, but with slightly inferior performance metrics, except for the sensitivity of the LR model, which reaches sensitivity values up to 82.4%. Finally, discarding some of the noisy data points results in a boost of the performance for all classifiers, with the respective performance metrics for the SVM model ranging from 81.4%-81.9%.

**Table 4:** Accuracy, sensitivity and specificity for the LR, RF, KNN and SVM classification models using the original and reduced feature set from fused EDA, HRV and ST data

	Method							
	*LR*		*RF*		*KNN*		*SVM*	
*Metric*	*Original*	*Reduced*	*Original*	*Reduced*	*Original*	*Reduced*	*Original*	*Reduced*
*Accuracy*	72.1%	79.6%	69%	78.7%	69%	77.2%	68.6%	81.6%
*Sensitivity*	76.8%	82.4%	65.6%	81.1%	65.7%	78.4%	59.2%	81.4%
*Specificity*	68.2%	76.5%	71.8%	76.2%	71.8%	76%	76.7%	81.9%

## Discussion

In this study, we investigated the potential of utilizing physiological data for the detection of adult ADHD. Specifically, we focused on three physiological signals: EDA, HRV, and ST, acquired using a wrist-worn sensor during a series of Stroop tests. Our main hypothesis was that these tests would elicit ANS responses that differ between neurotypical adults and adults with an ADHD condition. One of our primary objectives was to evaluate the performance of unimodal models using each physiological signal separately. Additionally, we explored the potential of the complementarity of information, provided by these data modalities and constructed a multimodal model combining them simultaneously, to enhance the overall model capabilities. Using our proprietary data processing infrastructure, we extracted a series of features for each physiological signal and employed them in ML algorithms for a classification task aimed at identifying neurotypical and ADHD populations. Our results supported that these physiological signals carry significant information to be correlated with underlying ADHD conditions. Specifically, we developed multimodal ML models that achieved up to approximately 82% sensitivity and specificity. In the following, we will delve into the interpretation and implications of these results.

Early in our investigation, we explored the ANS responses, as captured by the available physiological data, across the six different Stroop tests, and between the two participant groups. The aim was to identify the subset of our data that could yield optimal separability between the ADHD and the neurotypical populations. Initially, we analyzed each of the two participant groups separately. Through the use of Wilcoxon signed-rank tests, we found that each type of Stroop test (C, N and E) yields sufficiently distinct responses. More specifically, within the three Color-Word and the two Number Stroop tests, the average variation of the feature distributions was significantly lower than comparing feature distributions from different types of Stroop tests (i.e. Color-Word vs Number, Number vs Emotion and Color-Word vs Emotion). Therefore, for the Color-Word and Number Stroop tests, the impact of the specific test at the individual level was minimal, and thus, we merged the corresponding data under a single unique test label. Utilizing these new groupings, we gained a second insight regarding the efficiency of the Stroop tests in triggering ANS responses sufficient to distinguish between the two groups. Our results suggest that the Number Stroop tests yield far more features showing a statistically significant ($P\le .1$) difference between the CG and EG, than Color-Word or Emotion Stroop tests. In more detail, for the Number Stroop, approximately 52% of the total features exhibit a significant difference, while for the Color-Word and the Emotion Stroop, approximately only 10.4% and 3.6% of the total features, respectively, show significant differences. What is more, the percentage of statistically significant features is similar across the different data modalities, indicating their potential value in distinguishing between the CG and the EG. Specifically, statistically significant differences were primarily observed in the statistical time domain and morphological features of the EDA (e.g. mean, standard deviation, first difference, number of EDA responses, EDA responses characteristics, etc.), time and frequency domain features of the HRV (e.g. SDNN, RMSSD, pNN50, power in low and high frequency bands, etc.) and the time domain features of the ST (e.g. standard deviation, first and second difference, etc.).

The sensitivity of the Number Stroop test to group differences in ANS response compared to other Stroop tasks is an intriguing finding that warrants further discussion. This phenomenon can potentially be attributed to the inherent complexity of the numerical Stroop task, which involves comparing two stimuli, the numerical value, and the physical size, exhibiting higher cognitive load and complexity than the color-word Stroop task [100]. Therefore, higher ANS activation is expected during numerical tasks since they require substantial working memory and executive function resources [101]. Additionally, previous research suggests that the processing of numerical and physical magnitudes relies on a semantic abstract and non-verbal magnitude representation, which may facilitate or interfere with cognitive processes without the confounding effects of reading skill and articulatory speed commonly associated with ADHD [102]. Consequently, numerical Stroop tasks may be more effective in triggering cognitive challenges associated with ADHD conditions. Finally, the nature of the stimuli in the numerical Stroop task, where both relevant and irrelevant tasks involve the comparison of magnitudes, may lead to similar brain activations and require constant active control mechanisms to inhibit the irrelevant task [103–105]. As a result, it may be more difficult to modulate the task conflict, leading to increased ANS activation.

A later step of our analysis identified data points carrying significant information for their respective classes. This data mining procedure [106] has become essential when dealing with real-world data [106] and aims to filter out the least informative data points, thereby improving input quality for prediction models [106]. In this study, we specifically considered outlier data points from the neurotypical population. Given that participants in the CG were not previously assessed for potential ADHD symptomatology, ADHD-like characteristics in their physiological data might have emerged during the Stroop tests, despite their labeling as CG. In contrast, participants in the EG underwent assessment by a clinical expert, during which their inclusion in the EG group was verified, and, thus, their physiological expressions were representative of an ADHD condition. Among various methods for data filtering [106], we employed an extension of the k-Nearest Neighbors algorithm (i-KNN), which incorporates a distance metric that considers the class labels of the neighboring points [97, 98]. The impact of this filtering method was assessed by comparing the performance of prediction algorithms separately for the full and the reduced dataset. Based on the nature of the i-KNN algorithm, we anticipated a larger impact on prediction algorithms exploiting the geometrical properties of data [106]. To demonstrate the effect on the prediction capabilities of the different models, we utilized balanced accuracy, defined as the mean value of sensitivity and specificity. Our results indicate a consistently positive mean relative change in balanced accuracy across all signals separately and their fusion when using the reduced dataset. Notably, the relative change is more pronounced for the RF (15.21%), KNN (14.23%), and SVM (17.72%) models, which leverage the high-dimensional structure of the data. In contrast, the LR models, which rely on statistical properties, exhibit a smaller mean relative change (9.57%). Therefore, the rest of this section focuses on the reduced dataset.

**Fig. 6:**
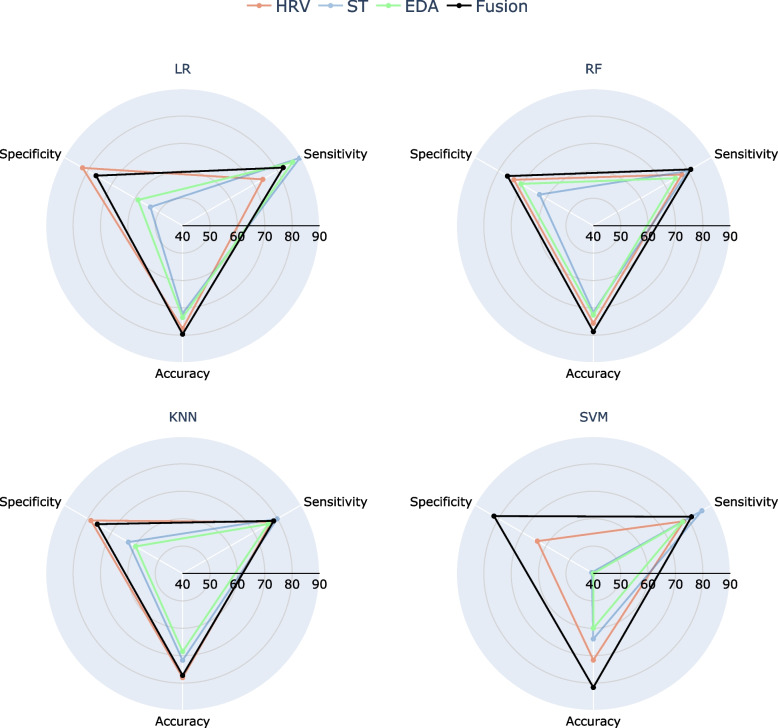
Test accuracy, sensitivity and specificity of the LR (top left), RF (top right), KNN (bottom left) and SVM (bottom right) models for EDA (orange line), HRV (light blue line), ST (light green line) and Fusion (black line), when the reduced dataset is utilized

The primary focus of this work was to explore the performance of models leveraging each physiological signal separately (i.e., unimodal), as well as the fusion of them (i.e. multimodal). In the following, we discuss the findings regarding the performance of these models and compare their effectiveness. Starting from the unimodal models, significant variations in performance were observed across different physiological signals and performance metrics. Figure 6 illustrates the accuracy, sensitivity, and specificity of each physiological signal and their fusion for each tested algorithm, focusing on the reduced dataset. Notably, EDA and ST exhibit equal or better sensitivity compared to HRV, while HRV shows higher specificity. The most interesting observation occurs when the fusion of signals is utilized. In this case, more complex models can be built by exploiting information from all signals simultaneously. Therefore, multimodal models demonstrate more balanced performance, also achieving higher sensitivity and specificity across all algorithms except for KNN (for the HRV dataset), compared to the unimodal ones, which exhibited either high sensitivity or high specificity. This suggests that combining information from multiple signals simultaneously improves model performance, highlighting the importance of considering multimodal approaches in such studies. Independent of the classification algorithm, we can argue that multimodal information from EDA, HRV and ST yields at least equal or better performance than unimodal for both neurotypical and ADHD adults. This could be attributed to the fact that the selected data modalities convey information about various aspects of the ANS [5, 13, 61, 63–66] and better capture the multifaceted effect of ADHD in autonomic functioning [107]. Further investigation into the underlying mechanisms driving these observations could provide valuable insights into the pathophysiology of ADHD and inform the development of more effective diagnostic and therapeutic strategies.

**Fig. 7:**
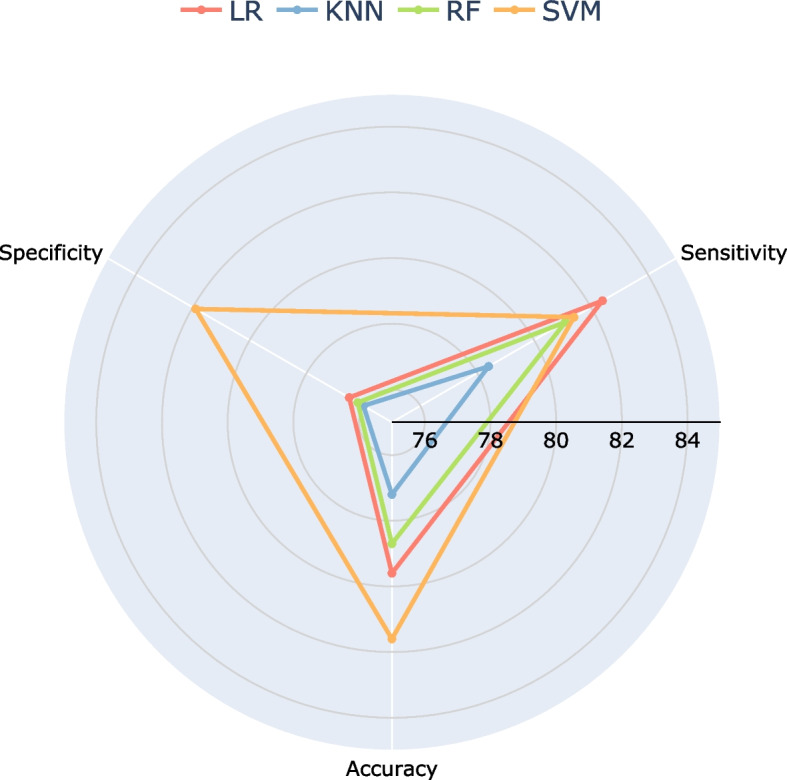
Test accuracy, sensitivity and specificity for the LR (red line), RF (green line), KNN (blue line) and SVM (orange line) models, when using all data modalities

Focusing on the multimodal models, a comparison across different algorithms was conducted to evaluate their performance. In Fig. 7, we present the accuracy, sensitivity, and specificity obtained for the LR, RF, KNN, and SVM algorithms for the multimodal case. Our analysis reveals that the SVM algorithm consistently outperforms the others, particularly in terms of accuracy and specificity. Although it is only slightly worse than LR in terms of sensitivity, SVM achieves the most balanced result overall. The superior performance of SVM in this context can be attributed to its ability to effectively utilize multimodal data and learn from its complex structure. By optimizing the utilization of multimodal information, SVM demonstrates proficiency in identifying both neurotypical and ADHD adults.

Overall, the findings of this study validate our hypothesis regarding ANS responses and their association with an underlying ADHD condition. Specifically, we demonstrated that ANS responses triggered by neuropsychological evaluations and captured by the EDA, HRV and ST physiological signals can effectively distinguish between ADHD and neurotypical adults. The most promising results were observed when following a multimodal data approach combining all signals captured during the execution of the Number Stroop tests. The SVM classification model exhibited the most balanced performance with regards to sensitivity and specificity (81.4% and 81.9%, respectively) highlighting the potential to recognize both ADHD and neurotypical adults. Future studies could further explore the potential of integrating additional sensor-collected data modalities (e.g. ambient conditions, accelerometer data, etc.), along with participants’ performance metrics from the Stroop tests (e.g. correct and wrong responses, response times, etc.) in the ADHD detection process. Academic research has recognized the potential contribution of these data sources to improve the efficiency of early ADHD diagnosis, but very limited clinical evidence is available [58].

Our findings contribute to addressing the lack of biomarkers for the detection of complex conditions like ADHD [24, 108]. Even though the current diagnostic process for adult ADHD has been widely used for decades, several limitations including accessibility, subjectiveness of the diagnosis, comorbidities, time and financial requirements, may hinder its efficiency and effectiveness. By leveraging passively collected multimodal physiological data, our study provides valuable insights into the pathophysiology of ADHD and presents a scalable solution for ADHD screening. The unobtrusive data collection method combined with the low cost of associated equipment render our platform a promising tool for assisting specialists during ADHD screening, supplementing traditional and resource-intensive methods. Furthermore, the use of physiological data obtained from wrist-worn devices opens avenues for continuous evaluation, enabling clinicians to monitor symptoms progression in near-real-time and obtain a more accurate clinical image of patients. This continuous monitoring could also lead to timely interventions and improved patient outcomes.

### Limitations

While this study provides valuable insights into ADHD detection using multimodal physiological data, a few study limitations should be acknowledged. The relatively small sample size restricts the complexity of the ML models built and the robustness of results obtained, highlighting the need for larger samples to enable more comprehensive analyses. This work relies on in-sample model validation methods (i.e. cross-validation), which may lead to over-optimistic performance estimates. Larger sample sizes will enable the utilization of out-of-sample validation methods, fortifying the generalizability and robustness of the study outcomes. Additionally, the study did not control for co-occurring disorders and medication usage among participants, potentially confounding the observed ANS responses and ADHD detection accuracy. Future research should address these confounding factors to improve the validity of findings. Moreover, the study focused on detecting ADHD as a general condition and did not differentiate between ADHD subtypes, warranting exploration of subtype-specific detection methods. Furthermore, incorporating a wider range of continuous performance tests beyond Stroop tests targeting other ADHD-related characteristics could enhance performance, while also improving the generalizability of findings. Finally, confirming the profiles of the control group participants through clinical evaluations, in order to ensure that only neurotypical individuals are included, would further enhance our understanding of the potential of ADHD detection using physiological data.

## Conclusion

In conclusion, this study has shed light on the potential of utilizing physiological data, including EDA, HRV, and ST for the detection of adult ADHD. By investigating ANS responses elicited during Stroop tests, we have demonstrated significant differences between neurotypical adults and those with ADHD, supporting the feasibility of using physiological signals as biomarkers for ADHD detection. Our analysis revealed that multimodal models, combining information from all physiological signals, outperformed unimodal ones, highlighting the importance of considering multimodal approaches in ADHD research. The SVM classification model emerged as the most effective in distinguishing between ADHD and neurotypical adults, achieving a balanced performance in terms of sensitivity and specificity (81.4% and 81.9%, respectively).

However, the relatively small sample size and the use of in-sample model validation methods pose challenges to the generalizability of our findings. Future research should aim to address these limitations by incorporating larger and more diverse samples, as well as employing out-of-sample validation methods to enhance the robustness of the results. Despite these limitations, our findings contribute to the growing body of literature on ADHD detection and underscore the potential of physiological data as valuable tools in clinical practice. Moving forward, further investigation into the underlying mechanisms driving ANS responses in ADHD and the integration of additional data modalities could provide deeper insights into the pathophysiology of the disorder and inform the development of more effective diagnostic and therapeutic strategies.

## Data Availability

The original contributions presented in the study are included in the article; Further inquiries can be directed to the corresponding authors.

## Abbreviations

ADHD: Attention-deficit/hyperactivity disorder
ANS: Autonomic nervous system
AQ: Autism-spectrum quotient
BAARS: Barkley adult ADHD rating scale–IV
CG: Control group
CPT: Continuous performance test
DIVA: Diagnostic interview for ADHD in adults
DPMP: Digital precision medicine platform
DSM-5: Diagnostic and statistical manual of mental disorders
EDA: Electrodermal activity
EEG: Electroencephalography
EG: Experimental group
EQ: Empathy quotient
HR: Heart rate
HRV: Heart rate variability
i-KNN: Informative KNN
KNN: k-Nearest neighbors
LR: Logistic regression
M.I.N.I.: Mini-international neuropsychiatric interview
MRI: Magnetic resonance imaging
PCA: Principal component analysis
RF: Random forests
rIQR: Relative interquartile range
rMAD: Relative median absolute deviation
ST: Skin temperature
SVM: Support vector machines
